# Effect of Cardiac Arrest on Cognitive Impairment and Hippocampal Plasticity in Middle-Aged Rats

**DOI:** 10.1371/journal.pone.0124918

**Published:** 2015-05-01

**Authors:** Charles H. Cohan, Jake T. Neumann, Kunjan R. Dave, Aleksey Alekseyenko, Marc Binkert, Kenneth Stransky, Hung Wen Lin, Carol A. Barnes, Clinton B. Wright, Miguel A. Perez-Pinzon

**Affiliations:** 1 Cerebral Vascular Disease Research Laboratories, University of Miami Leonard M. Miller School of Medicine, Miami, Florida, United States of America; 2 Evelyn F. McKnight Brain Institute, University of Miami Leonard M. Miller School of Medicine, Miami, Florida, United States of America; 3 Department of Neurology, University of Miami Leonard M. Miller School of Medicine, Miami, Florida, United States of America; 4 Neuroscience Program, University of Miami Leonard M. Miller School of Medicine, Miami, Florida, United States of America; 5 Evelyn F. McKnight Brain Institute; ARL Division of Neural Systems, Memory & Aging; Departments of Psychology, Neurology and Neuroscience, University of Arizona, Tucson, United States of America; Georgia Regents University, Medical College of Georgia, UNITED STATES

## Abstract

Cardiopulmonary arrest is a leading cause of death and disability in the United States that usually occurs in the aged population. Cardiac arrest (CA) induces global ischemia, disrupting global cerebral circulation that results in ischemic brain injury and leads to cognitive impairments in survivors. Ischemia-induced neuronal damage in the hippocampus following CA can result in the impairment of cognitive function including spatial memory. In the present study, we used a model of asphyxial CA (ACA) in nine month old male Fischer 344 rats to investigate cognitive and synaptic deficits following mild global cerebral ischemia. These experiments were performed with the goals of 1) establishing a model of CA in nine month old middle-aged rats; and 2) to test the hypothesis that learning and memory deficits develop following mild global cerebral ischemia in middle-aged rats. To test this hypothesis, spatial memory assays (Barnes circular platform maze and contextual fear conditioning) and field recordings (long-term potentiation and paired-pulse facilitation) were performed. We show that following ACA in nine month old middle-aged rats, there is significant impairment in spatial memory formation, paired-pulse facilitation n dysfunction, and a reduction in the number of non-compromised hippocampal Cornu Ammonis 1 and subiculum neurons. In conclusion, nine month old animals undergoing cardiac arrest have impaired survival, deficits in spatial memory formation, and synaptic dysfunction.

## Introduction

Out-of-hospital cardiac arrest (CA) is a leading cause of death and disability in the United States, affecting approximately 400,000 people annually [1]. Survival rates after out-of-hospital CA have remained unchanged at 8% over the past three decades despite improved resuscitation techniques and emergency therapies (i.e. hypothermia) [2–5]. Numerous epidemiological studies have indicated that the risk of CA increases as an individual ages [2, 6–8]. As a consequence, the Stroke Therapy Academic and Industry Roundtable on preclinical research recommended testing putative neuroprotective therapies using aged animal models [9].

CA induces global ischemia, disrupting global cerebral circulation, which can induce neuronal damage in numerous brain regions including the hippocampus, cortex, cerebellum, thalamus, prefrontal cortex, and putamen [10–12]. Survivors of CA experience cognitive deficits including dysfunction in memory, psychomotor speed, and executive function [12–14]. Various animal models of global cerebral ischemia have also suggested damage to these brain regions with concomitant cognitive deficits [15–18]. The Cornu Ammonis 1 (CA1) region of the hippocampus is particularly sensitive to cerebral ischemia and a common region investigated for neuronal cell death [10, 19]. This damage is of particular concern as the hippocampus is an important player in memory consolidation and individuals with hippocampal damage suffer from anterograde amnesia [20]. To date, the primary methods to induce global cerebral ischemia have relied upon two-vessel occlusion (2-VO) [21–23] and four-vessel occlusion (4-VO) [24–26] models. Although these techniques result in cortical and hippocampal damage, they lack a cardiac component, which may be a contributing factor to developing cognitive dysfunction over time [27]. Additionally, the 4-VO model is only partially reversible. Previous studies that have used a CA model have highlighted spatial memory deficits following ischemia in young rodents [15, 28]; however, a middle-aged animal has not been investigated for spatial memory deficits, despite the observation of hippocampal cell death [29].

Currently, the majority of studies investigating CA have used animal models that utilize juvenile animals (commonly 2–4 months) [30–33]. One major discouraging factor for investigators using aged animal models has been poor post-CA survival. For example, Xu *et al*. found that the four day survival in 24 month old rats was 40% after seven minutes of CA [34]. However, despite this survival disadvantage, performing CA using aged animals may be a more clinically relevant model in determining the efficacy of putative agents for neuroprotection that will be used in clinical trials [35, 36]. In this current study, we used a model of asphyxial CA (ACA) to induce global cerebral ischemia in nine month old male Fischer 344 rats in order to investigate the effects of mild global cerebral ischemia on middle-aged animals. The goals of this study were to 1) establish a model of ACA in middle-aged rats and 2) to test the hypothesis that learning and memory deficits develop following mild global cerebral ischemia in middle-aged rats.

## Materials and Methods

### Animals

All animal procedures were performed in accordance with the Guide for the Care and Use of Laboratory Animals published by the National Institutes of Health and approved by the Animal Care and Use Committee of the University of Miami. Animals were separated into either sham (n = 22) or asphyxial cardiac arrest (ACA; n = 45) groups for each experiment. Only sham (n = 21) or CA (n = 17) animals that survived 7 days post-surgery were used for data analysis. Animals that underwent histological analysis (11 total, 6 sham, 5 ACA) did not undergo Barnes maze or fear conditioning testing due to the ability of enriched environment to increase cell survival post-ischemic injury [37]. Animals that underwent long-term potentiation (LTP; 12 total, 6 sham, 6 CA) testing were trained on a Barnes maze in order to reduce any variability caused by exposure to the Barnes maze and did not undergo fear conditioning as fear conditioning can impair LTP induction [38]. Remaining animals were used for Barnes maze testing and fear conditioning.

### Induction of Cardiac Arrest

ACA was induced as previously described [30, 31]. Male Fischer 344 rats (nine month old) weighing 430.2 ± 3.5 g were fasted overnight and then anesthetized with 4% isoflurane and 30:70 mixture of O_2_ and N_2_O by inhalation. The femoral artery was cannulated for blood pressure measurements and arterial sampling of blood gases. Arterial blood gases (Radiometer, Copenhagen, Denmark) and plasma glucose levels (One Touch glucose monitor; LifeScan, Milpitas, CA) were measured throughout the experiment. Head and body temperature were maintained at 37°C throughout the experiment. Rats were immobilized throughout the procedure with vecuronium bromide (2.0 mg/kg, intravenous [IV], administered every 10 minutes). ACA was induced through apnea by disconnecting the ventilator from the endotracheal tube. Six minutes after asphyxia, resuscitation was initiated by administering a bolus injection of epinephrine (0.005 mg/kg, IV) and sodium bicarbonate (1 meq/kg, IV) followed by mechanical ventilation of 100% O_2_. Sham animals were subjected to similar surgical procedures without the induction of asphyxia, resuscitation drugs, and mechanical ventilation of 100% O_2_. Following ACA or sham procedures, animals were monitored daily for body weight, rectal temperature, hydration, and blood glucose. If needed, animals were gavage-fed with liquefied animal chow, injected with saline, and/or maintained in a humidified warm incubator.

### Histology

Seven days after sham or ACA procedures, rats were anesthetized with isoflurane, perfused with physiologic saline for one minute, and then perfused for 19 minutes with FAM (a mixture of 40% formaldehyde, glacial acetic acid, and methanol, 1:1:8 by volume). The perfusate solution was delivered into the root of the ascending aorta at a constant pressure of 110–120 mmHg, as previously described [39]. The head was removed and immersed in FAM for one day at 4°C before the brains were removed from the skull and gross sectioned using a rat brain matrix (ASI instruments, MI, USA). Coronal brain blocks were processed in a tissue processor (Leica TP1050, Germany) and embedded in paraffin using a Histo-Center-II embedding station (Fisher Scientific, PA, USA). Coronal brain sections (10 μm) were cut using a high profile Teflon coated disposable blade (Ted Pella, CA, USA) on a rotatory microtome (Leica RM2135). Brain sections were then placed on a glass slide and paraffin was removed by incubating overnight in an oven at 54 ± 1°C. The sections were then stained with hematoxylin (Gill’s Formulation #2, Fisher Scientific, PA, USA) and eosin (0.5% eosin solution in 80% ethanol and 0.5% glacial acetic acid). Neuronal counts (“non-compromised” neurons) were made by an investigator blinded to the experimental conditions within the CA1 and subiculum regions using three coronal brain sections 200 μm apart starting at the level of -3.8 mm from bregma. Cell counts were average per hemisphere and field of view.

### Acute Slice Preparation

Hippocampal slices were prepared from rats seven days following ACA [40]. Animals were anesthetized with 100 mg/kg ketamine and 10 mg/kg xylazine intraperitoneal and placed on ice. Animals were then transcardially perfused with a sucrose artificial cerebrospinal fluid (aCSF) (in mM: sucrose, 206; KCl, 2.8; CaCl_2_, 1.0; MgCl_2_, 1.0; MgSO_4_, 2.0; NaHCO_3_, 26; Na_2_HPO_4_, 1.25; ascorbic acid, 0.4; glucose, 10; and oxygenated with 95% O_2_ – 5% CO_2_) at 4°C that contained 20 μM 6-cyano-7-nitroquinoxaline-2,3-dione and 20 μM R-2-amino-5-phosphonopentanoate. The animals were decapitated, the brain was rapidly removed. Coronal slices of 400 μm in thickness were sectioned using a Leica VT1000S microtome in sucrose aCSF at 4°C and transferred into a solution containing 50% sucrose aCSF/50% aCSF at room temperature for 30 min before being transferred/stored in aCSF (in mM: NaCl, 126; KCl, 3.5; CaCl_2_, 2.0; MgSO_4_, 2.0; NaHCO_3_, 26; Na_2_HPO_4_, 1.25; sodium ascorbate, 0.4; glucose, 10; and oxygenated with 95% O_2_–5% CO_2_) at room temperate. Individual slices were then transferred to an interface recording chamber (BSC-BU chamber, Warner Instruments, Hamden, CT, USA), superfused with warmed (33 ± 1°C) aCSF (TC-344C, Warner Instruments) at a rate of 1 ml/min, and oxygenated with humidified 95% O_2 –_ 5% CO_2_ [30].

### Electrophysiology

General population measurements of field excitatory postsynaptic potentials (fEPSP) were recorded with NaCl-filled (150 mM) glass micropipettes inserted into the stratum radiatum of the CA1 hippocampal subfield using a SUPER-Z head-stage attached to a BMA-931 AC/DC Bioamplifier (CWE-inc, Ardmore, PA, USA), Digidata1200 (Molecular Devices, Sunnyvale, CA, USA), and pClamp 9.0 software (Molecular Devices). Schaffer collaterals were electrically stimulated (0.3 ms constant current pulses) with bipolar tungsten electrodes using a S48 square pulse stimulator (GRASS technologies, Warwick, RI, USA). After slice recovery in the recording chamber (approximately 30 min), input–output curves relating stimulus current intensity to fEPSP slope and amplitude were generated. Stimulus intensity required for half-maximal fEPSP slope was selected. Paired-pulse experiments were performed using an intra-pulse duration of 50 ms. Stimulation for fEPSP was performed at a rate of 1 stimulus every 30 seconds for baseline recordings. Long-term potentiation (LTP) was then induced using tetanic stimulation (100 Hz) for 1 second. One minute following LTP, test stimulation (1 stimulus every 30 seconds) resumed for a period of 1 h and afterwards input–output curves were repeated. Slices from each animal were averaged together to give a n-value of one.

### Barnes Circular Platform Maze

The Barnes circular platform maze is a less physically demanding alternative to the Morris water maze [41], which is used to evaluate hippocampal dependent spatial memory deficits [42]. Animals were tested three days after ACA for four consecutive days; however, to acclimate the animals to human touch, each rat was handled for three days (10 min session) prior to ACA induction. Testing was performed just after onset of the dark cycle for the rats. The Barnes circular platform maze is a 122 cm diameter circular platform on a 1.4 m stand with 18 evenly spaced 9.5 cm diameter holes around the circumference, where a black box (escape tunnel) was placed underneath one of the holes (Med-Associates Inc., St. Albans, VT, USA). Four bright lights were positioned above the platform as an aversive stimulus and cause the rat to seek out the escape tunnel using spatial cues. Prior to the first trial, animals were subjected to a habituation trial by placing the rat next to and allowing the rat to enter the escape tunnel for one minute before being returned to its home cage. On all subsequent trials, the animals were placed into the center of the apparatus under a dark container for 30 seconds before the container was lifted and the rat was allowed to navigate the maze using spatial cues surrounding the apparatus (images). Each rat was allowed 180 seconds to locate and enter the escape tunnel per trial. If the rat was unable to locate the escape hole after 180 seconds, the rat was gently guided to the correct hole location and allowed to enter the escape tunnel. Once the rat entered the escape tunnel (either guided or on their own), it remained in the tunnel for two minutes before returning to its home cage. Each day, the animal would attempt two trials spaced 15 minutes apart for a total of eight trials. In between each trial, the platform and escape tunnel were cleaned with 70% ethanol and water. Video recordings were made using an EQ610 Polestar II Everfocus camera. The latency to entry into the escape tunnel and distance traveled were quantified using Ethovision 8.5 video tracking software (Noldus, Leesburg, VA, USA).

### Search Strategy

The search strategy for each animal was analyzed in a manner adapted from previously published methods [43, 44]. Briefly, each Barnes maze trial was classified into one of three search strategies: random, serial, or spatial. Use of a spatial strategy indicates use of cues in a spatial manner in order to locate the target quadrant. The strategy was classified as “spatial” if from the start of the trial the animal entered the correct quadrant and made two or less errors before entering the escape tunnel. Use of a serial strategy indicates a systematic, non-random method for locating the target hole independent of spatial cues. The strategy was classified as “serial” if the rat circled around the outside of the maze in a systematic fashion until the escape tunnel was located and entered. The use of a random search strategy indicates a non-systematic, non-spatial method for locating escape tunnel, indicating an inability to develop a cognitive strategy to locate the hole. The strategy was classified as “random” if the rat looked in one or more incorrect hole(s) then crossed the midline of the maze more than once. After classification of search strategies, two separate analyses were conducted to compare search strategy utilization differences between sham and ACA animals. The first analysis examined the ability of the rats to use spatial cues. The number of trials where animals used a spatial strategy versus a non-spatial strategy (i.e. systematic or random strategy) was counted; differences between sham and ACA animals were compared. The second analysis examined use of non-random search strategy. The number of trials where systematic non-random search strategies (i.e. spatial or serial) versus non-systematic, random search strategy (i.e. random strategy) was quantified; again, differences in utilization between sham and ACA animals were compared. Comparisons were made using chi square analyses. Null hypothesis testing was conducted comparing the expected results if ACA had no effect on search strategies (i.e. the observed sham values), compared to the observed ACA search strategy values. Significance was determined at p < 0.05.

### Contextual Fear Conditioning

To determine if spatial memory deficits persisted 6–7 days after ACA, contextual fear conditioning was performed. Fear conditioning occurred in a conditioning apparatus (12" W x 10" D x 12" H) placed inside of an isolation cubicle (30” W x 17.75” D x 18.5” H) (Coulbourn instruments, Whitehall, PA). The isolation cubicle contained an overhead stimulus light and a 28 V exhaust fan that were left on during the trials. A drop pan was placed underneath an electrically active floor grid that was connected to a precision animal shocker (Coulbourn instruments, Whitehall, PA). Six days following ACA, after completing the Barnes circular platform maze, fear conditioning was induced. For this process, rats were placed into the testing room containing the conditioning apparatus for 30 minutes prior to testing. The conditioning apparatus was cleaned with 70% ethanol immediately prior to each trial. For the induction of fear, rats were placed into the conditioning apparatus for 340 seconds, at which time the animals receive a two second shock (1.5 mA). The rats would then spend an additional 28 seconds in the apparatus before completing the trial and returning to their home cage. On the second day, the animals were again returned to the room containing the conditioning apparatus again for 30 minutes prior to the trial. The rats were then placed into the apparatus for eight minutes with no shock to measure freezing behavior. Freezing behavior for both trials was quantified as the percent of time spent frozen using the visual tracking software FREEZEFRAME (Coulbourn instruments, Whitehall, PA).

### Statistics

All data are expressed as mean ± S.E.M. Statistical evaluation of the data was performed using one-way ANOVA, two-way ANOVA, or Student’s *t*-test when appropriate. For the search strategy measurements, a chi square analysis was used to compare between spatial and non-spatial search strategies and the random and systematic search strategies.

## Results

### Physiological Parameters

Before the induction of sham or ACA procedures, the physiological parameters were measured and no statistical differences were observed between groups (**Table 1**). Following surgery, the pO_2_ level in animals subjected to ACA (177 ± 16 mmHg, **p<0.01) was significantly higher than that of sham-operated animals (111 ± 5 mmHg), due to the delivery of 100% oxygen following restoration of spontaneous circulation (ROSC). In addition, the pH of the ACA animals was slightly reduced following ACA procedures. Also, the MABP in animals subjected to ACA (122 ± 2 mmHg) was significantly higher than that in sham-operated animals (107 ± 5 mmHg, ***p<0.001) potentially due to the use of epinephrine to initiate ROSC. All of the additional physiological parameters were not significantly different between sham or ACA procedures.

**Table 1:** Physiological parameters.

Groups	Variable	Asphyxial Cardiac arrest	
		Before	After
Sham	Body weight (grams)	434 ± 4	
	pH	7.47 ± 0.02	7.48 ± 0.01
	pCO_2_ (mm Hg)	35 ± 1	34 ± 1
	pO_2_ (mm Hg)	118 ± 6	111 ± 5
	Plasma glucose (mg/dl)	134 ± 5	
Asphyxial Cardiac arrest	Body weight (grams)	422 ± 7	
	pH	7.47 ± 0.01	7.39 ± 0.01***
	pCO_2_ (mm Hg)	37 ± 1	43 ± 2
	pO_2_ (mm Hg)	130 ± 6	177 ± 16**
	Plasma glucose (mg/dl)	138 ± 4	

**p<0.01

***p<0.001.

### Survival rates following ACA

**Fig 1:**
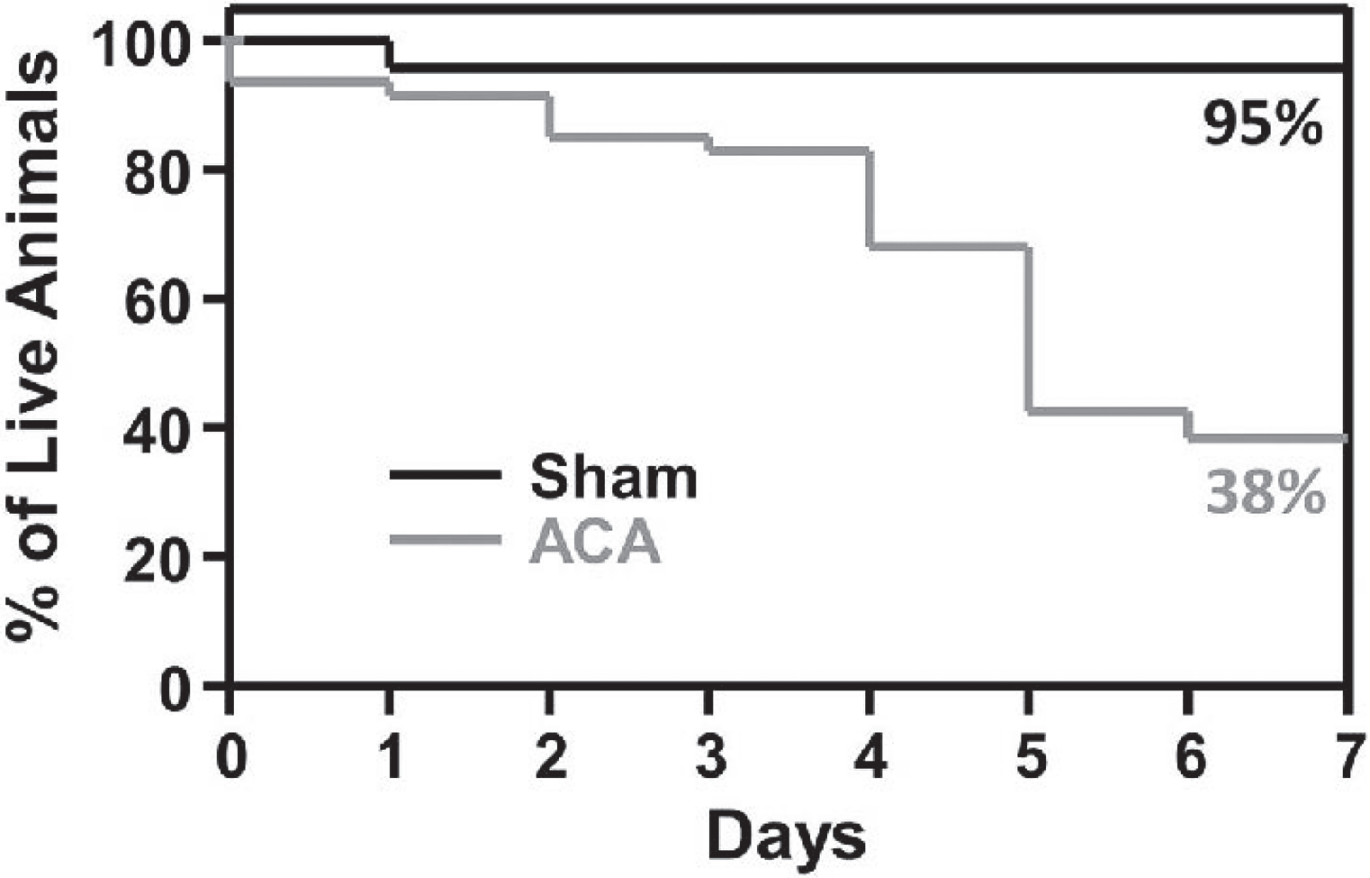
Survival rate following Sham or ACA procedure. Kaplan-Meier curve of animal survival for seven days following Sham or ACA procedures. At day seven, the survival of Sham animals was 95% and 38% for ACA rats (Sham n = 22 and ACA n = 45).

Following sham or ACA procedures, animal survival was observed for seven days. Survival for the first few animals that were exposed to ACA was limited, therefore, post-operative care was instituted and animals were monitored daily for body weight, rectal temperature, hydration, and blood glucose. If needed, animals were gavage-fed with liquefied animal chow, injected with saline, and/or maintained in a humidified warm incubator. Seven-day survival in animals subjected to sham surgery was 95%, where one sham-operated animal died following surgery (apparently due to lung dysfunction). While a majority of animals survived four days following ACA (83%), the overall seven-day survival in animals subjected to ACA was significantly reduced to 38% (**Fig 1**; p<0.05, Log-Rank test). Most of the animals subjected to ACA died of unknown complications; however, some deaths ensued from hypothermia, kidney failure, and/or lung edema.

### ACA decreased the number of non-compromised hippocampal CA1 and subiculum neurons

**Fig 2:**
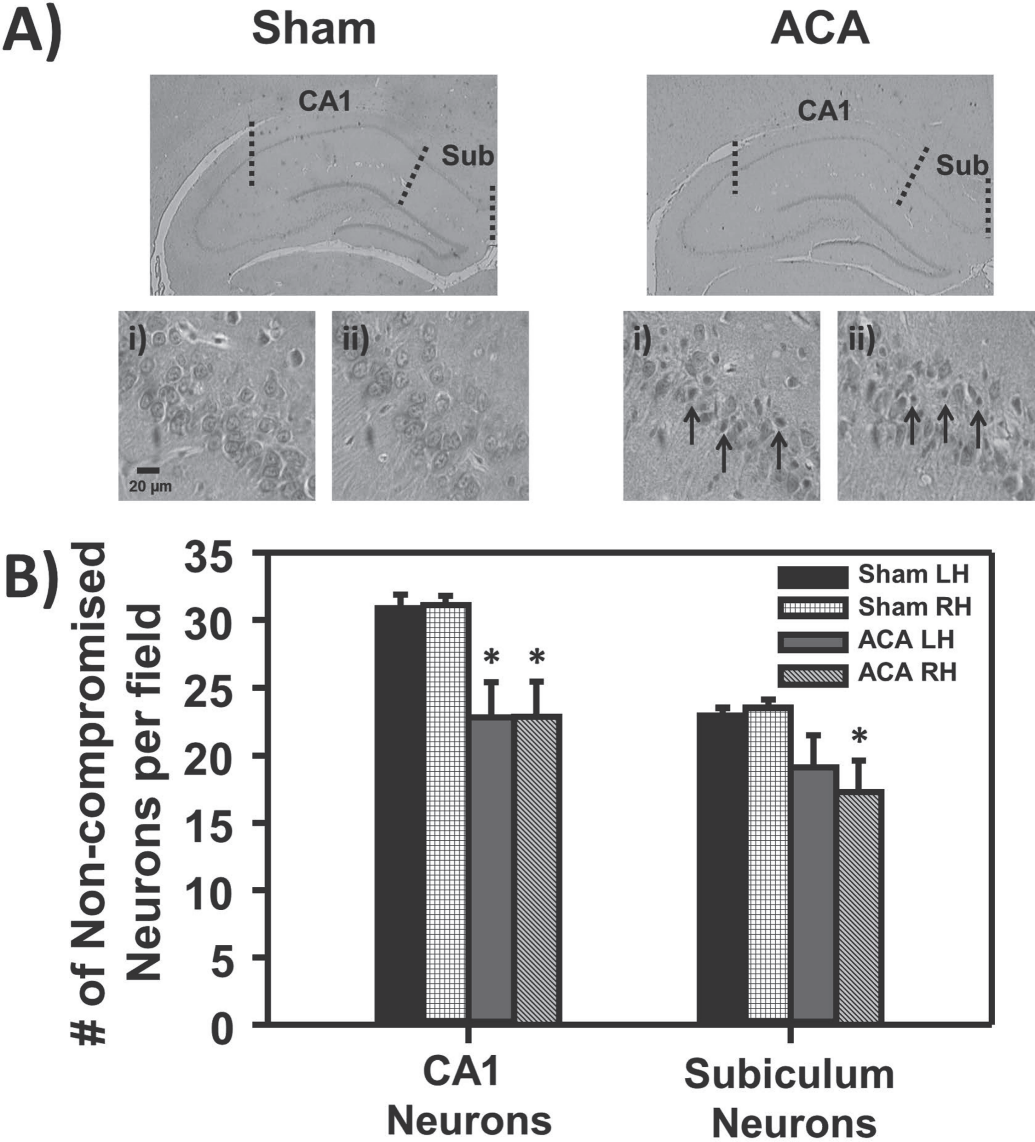
Histopathology of the hippocampus seven days following Sham or ACA. **A)** Representative histological images of hippocampal coronal slices of Sham and ACA animals with 40X images of the **i)** CA1 and **ii)** subiculum region. There was a visible increase in small pyknotic and eosinophilic cells (arrows) following ACA in the CA1 and subiculum regions. **B)** Bar graph depicting the number of non-compromised neurons per field to left (LH) or right (RH) hemisphere to their respective groups, where there was a significant decrease in the number non-compromised neurons following ACA in the CA1 region and RH of the subiculum (*p<0.05; Sham n = 6 and ACA n = 5).

Rats subjected to sham or ACA surgeries were sacrificed seven days after ACA for histopathology. Neurons with “non-compromised” characteristics (cells that do not exhibit ischemic cell change such as eosinophilic cytoplasm, dark-staining triangular-shaped nuclei, and eosinophilic-staining nucleolus) were counted in the CA1 region of the hippocampus and subiculum (**Fig 2A**). The number of non-compromised CA1 neurons in the hippocampus from the ACA group was significantly lower in both the left and right hemispheres compared to sham animals by 26% (**Fig 2B**; p<0.05; Sham n = 6 and ACA n = 5). Similarly, ACA-treated animals resulted in a 26% reduction of non-compromised neurons in the right hemisphere of the subiculum as compared to sham-operated rats (**Fig 2B**; p<0.05; Sham n = 6 and ACA n = 5). Conversely, the left hemisphere of the subiculum had a 17% reduction in non-compromised neurons, however this reduction was non-significant (**Fig 2B**; Sham n = 6 and ACA n = 5).

### ACA induced synaptic transmission dysfunction

**Fig 3:**
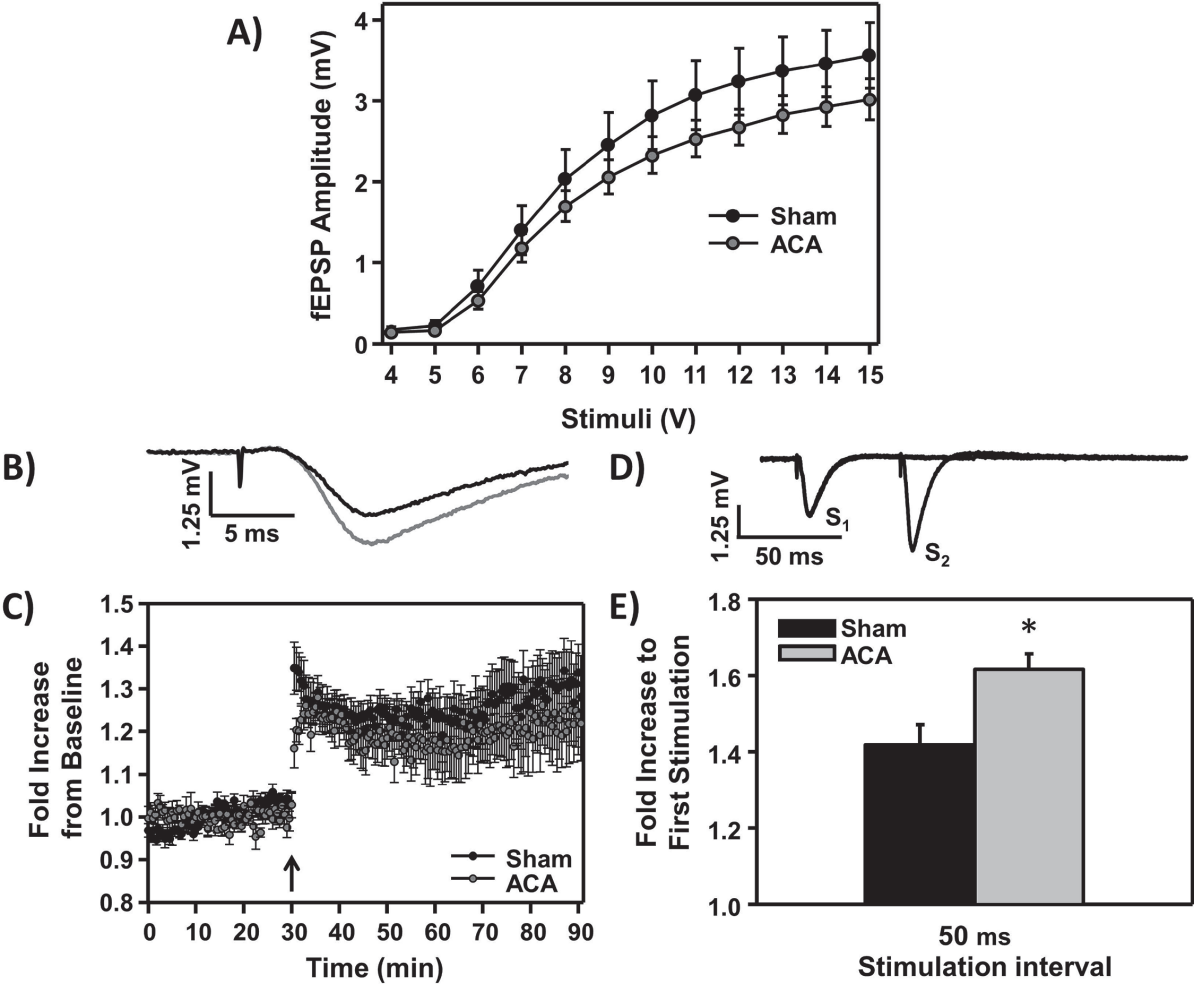
Hippocampal acute slice electrophysiology seven days following Sham or ACA procedure. **A)** Mean (± S.E.M.) input/output curve (I/O) of fEPSP amplitudes across stimulation intensities (4–15 V) in control and ACA animals (not normalized). **B)** Example tracings of sham fEPSP recorded in the CA1 stratum radiatum region of hippocampal slices, before (black) and after (gray) LTP-induction. **C)** Average slope of fEPSP (averaged to the 30 min pre-tetanus values) before and after the induction of LTP. LTP was induced by 100-Hz tetanic stimulation for 1 s and is indicated by the arrow. **D)** Example tracings of sham paired-pulse response. **E)** Average fold increase of paired-pulse response; Stimulation 2 (S_2_)/Stimulation 1 (S_1_) amplitude following sham and ACA procedures (*p<0.05, n = 6).

To investigate the synaptic activity in the stratum radiatum of CA1 hippocampal neurons following sham or ACA, hippocampal slices were harvested seven days following surgery and the induction of long-term potentiation (LTP) in CA1 neurons was analyzed. There was no statistical difference in the input/output curve between sham and ACA slices (**Fig 3A**) and both the stimulation intensity and half-max response were not significantly different between groups (**Table 2**). Using 50% maximum stimulation, baseline recordings were made every 30 sec for 30 min before and 60 min following tetanus stimulation (example sham LTP induction tracings, **Fig 3A**). The average slope calculated from each stimulus, normalized to the 30 min baseline recording, is shown in **Fig 3B**. Hippocampal slices from both sham and ACA-treated groups induced LTP following tetanic stimulation (**Fig 3B**; n = 6). Paired-pulse facilitation was investigated seven days following sham or ACA procedures (example of sham paired-pulse facilitation tracing, **Fig 3C**). There was a significant increase in the maximum amplitude of the paired-pulse response following ACA at the stimulation interval of 50 ms, where sham animals had a 1.42 ± 0.05 fold increase in response compared to 1.62 ± 0.04 fold increase following ACA (**Fig 3D**; n = 6, *p<0.05).

**Table 2:** Average stimulation intensity (V) required to induce 50% fEPSP amplitude (mV).

	Stimulation intensity (V)	50% fEPSP amplitude (mV)
**Sham**	8.54 ± 0.24	1.78 ± 0.20
**ACA**	8.55 ± 0.23	1.51 ± 0.13

### ACA induced spatial memory deficits on the Barnes circular platform maze

**Fig 4:**
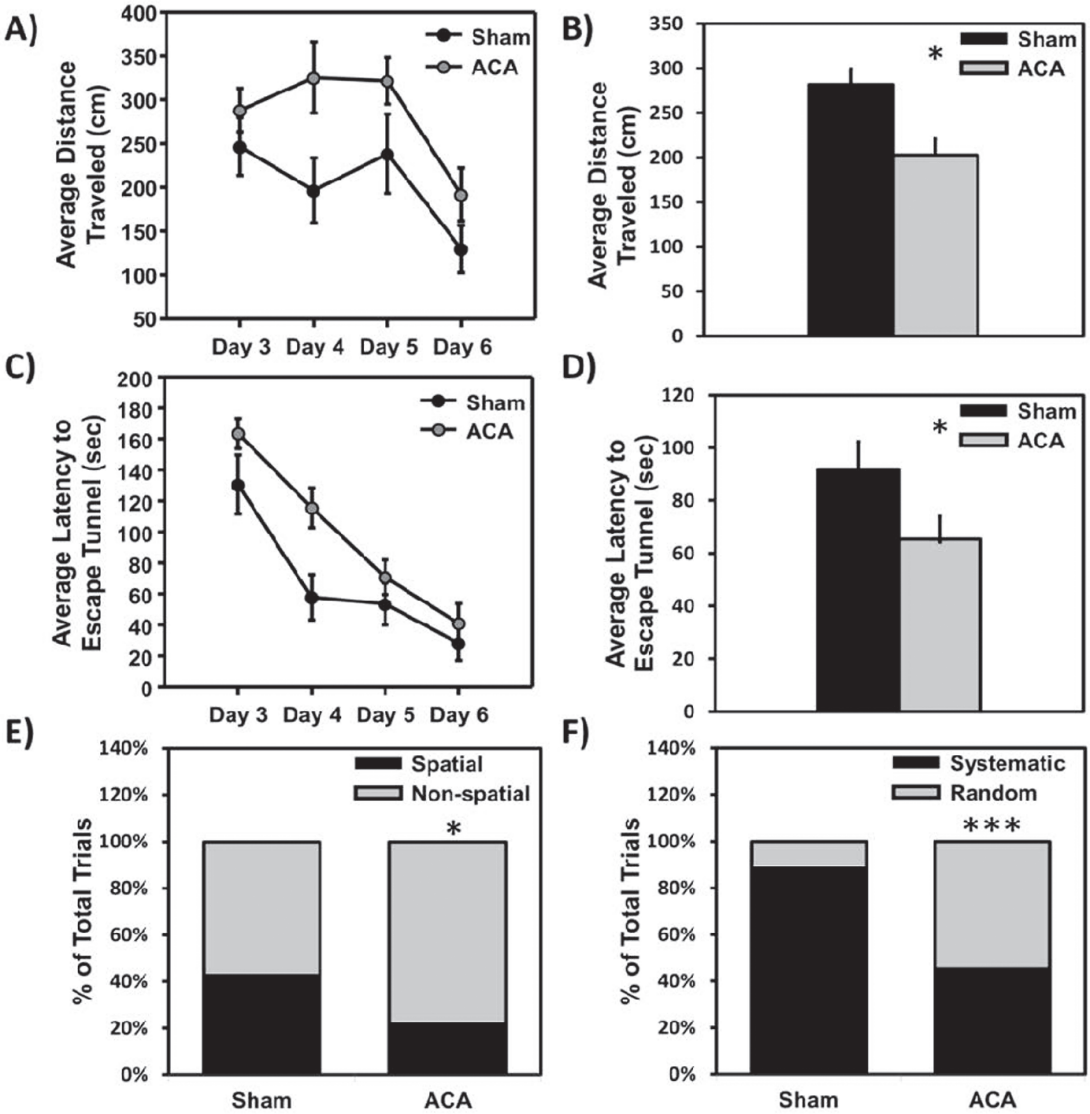
Barnes maze cognitive measurements following Sham or ACA procedure. Average distance traveled **A)** per day [F (1, 3) = 6.68, *p<0.05, group effect] or **B)** across all trials (**p<0.01) on Barnes circular platform maze (testing occurred 3, 4, 5, and 6 days following sham or ACA procedures). The average latency to enter escape tunnel **C)** per day (*p<0.05, group effect) or **D)** across all trials (***p<0.005, group effect) on Barnes circular platform maze. The percentage of each animal using a search strategy (spatial, serial, or random) was also quantified on the Barnes maze. **E)** Percentage of trials where an animal used a spatial versus a non-spatial (serial + random) search strategy (*p<0.05). **F)** Percentage of trials where an animal used a systematic (spatial + serial) versus a random search strategy (***p<0.005) (Sham n = 10 and ACA n = 8).

To correlate a decrease in non-compromised neurons (**Fig 2**) and paired-pulse facilitation dysfunction (**Fig 3**) with cognitive deficits, rats were tested using the Barnes circular platform maze to measure spatial memory deficits. The distance traveled and latency (time to enter the escape tunnel) were measured and averaged for each day as an indication of spatial memory deficits (**Fig 4;** n = 10 sham and n = 8 ACA). Both groups significantly improved in performance for both the distance traveled (**Fig 4A**) [F (1, 3) = 8.30, *p<0.05)] and latency (**Fig 4C**) [F (1, 3) = 6.68, *p<0.05] measured over four days. Additionally, there was a difference between the two groups observed in both distance traveled (represented in **Fig 4B**) [F (1, 3) = 6.07, **p<0.01), and latency [F (1, 3) = 35.69, ***p<0.005] (represented in **Fig 4D**). However, there was no significant surgery by time interaction when comparing the sham and ACA groups across the four day period for distance [F (3, 14) = 0.70] or latency [F (3, 14) = 1.62] measures.

### ACA treatment induced spatial search strategy and strategic deficits on the Barnes Circular Platform Maze

To further evaluate changes in memory and cognition, the search strategy used by each animal was evaluated for each trial on the Barnes maze (**Fig 4E & 4F**), (**S1 Table**). The percentage of ACA rats using a spatial search strategy (21.9%) was reduced compared to sham animals (42.5%) (Chi-Square, *p<0.05) (**Fig 4E**). Additionally, sham animals used a systematic non-random strategy (either serial or spatial) 78.1% of the time, compared to the ACA animals that used a systematic non-random search strategy 57.5% of the time (Chi-Square, ***p<0.005) (**Fig 4F**).

### ACA treatment induced a decrease in freezing behavior during contextual fear conditioning

**Fig 5:**
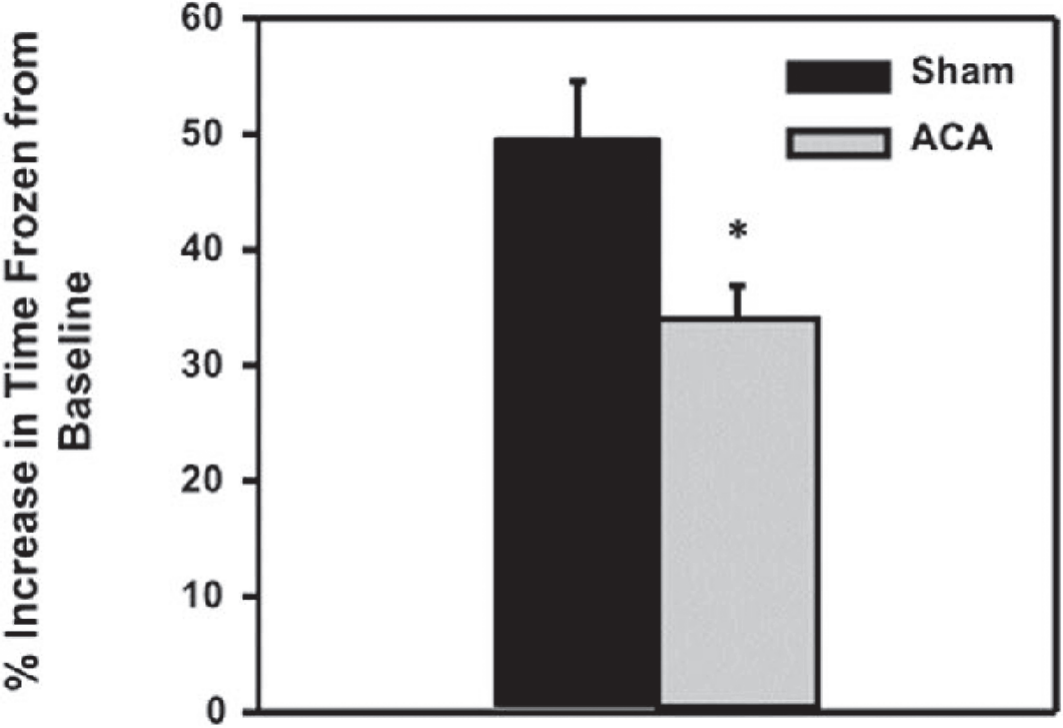
Fear conditioning spatial memory deficits following Sham or ACA procedure. Average increase in percent time frozen from baseline following fear conditioning was significantly higher in sham animals (51.44 ± 5.21%) compared to the ACA animals (33.97 ± 2.9%, *p<0.05) (Sham n = 9 and ACA n = 6).

In addition to the Barnes circular platform maze, contextual fear conditioning was completed to confirm spatial memory deficits seven days following ACA. Contextual fear conditioning was induced six days after sham or ACA procedures. Upon returning to the conditioning apparatus the following day, the percent increase in freezing behavior from baseline in sham animals was significantly higher (51.44 ± 5.21%) compared to the ACA animals (33.97 ± 2.90%, *p<0.05) (**Fig 5**; n = 9 sham and n = 6 ACA), suggesting that ACA-induced rats were unable to recall the context as compared to sham rats.

## Discussion

Global cerebral ischemia induces a variety of physiological alterations in the body, including neuronal damage and cognitive impairments [10, 19, 45]. As previously mentioned, numerous investigators in the field of cerebral ischemia have used 2-VO [21, 22] or 4-VO models [24–26], Additionally, previous studies have primarily used young animals (2–4 months) to investigate the resultant cognitive impairments and neuronal alterations due to experimental feasibility and low attrition after CA [30–33]. Using these models, several pharmacological agents have been suggested as putative therapeutics through the use of young animals [45]; however, the translational feasibility to the clinic is still very limited [35, 36]. Therefore, we sought to establish a model of CA and characterize potential memory deficits in middle-aged rats as a putative model for testing potential therapeutics against mild ischemia.

Our findings indicate a significant decrease in the number of non-compromised hippocampal CA1 and subiculum neurons seven days following ACA in nine month old male Fischer 344 rats as compared to age-matched shams. A previous report has described an observed increase in hippocampal cell death following cardiac arrest in 12 month old Fischer 344 rats as compared to 6 month old rats [34]; indicating that age may play a role in worse outcomes following cardiac arrest. In our study, the seven day survival following ACA in nine month old Fischer 344 rats was significantly reduced from 95% in the sham group to 38% in the ACA group (**Fig 1**). This decline in survival is greatly reduced compared to our previous studies in Sprague Dawley rats, where the seven day survival rates were around 80–90% at seven days [46, 47]. Ischemic events may be increasingly detrimental as an individual ages as compared to their younger counterparts, a trend that has been previously suggested [48, 49]. In our study, ACA-treated rats also exhibited impairment in spatial memory formation when measured using the Barnes circular platform maze and contextual fear conditioning. Furthermore, potential executive function impairments were observed through the increased use random search strategies in ACA rats. Additionally, there was an observed synaptic dysfunction characterized by the increase in paired-pulse facilitation following ACA.

Dysfunction in synaptic transmission occurs following cerebral ischemia [30, 50–54]. In this study, paired-pulse facilitation was measured seven days following ACA, where there was a significant increase in the maximal amplitude of the second stimulation fEPSP response (50 ms) compared to sham animals. Paired-pulse facilitation is dependent on presynaptic sequestration of Ca^2+^ to limit the amount of glutamate release during repetitive stimulation [55]; a reduction in the presynaptic clearance of Ca^2+^ can lead to an increase in glutamate release during short interval stimulation. This pre-synaptic dysfunction may be due to a deficit in Ca^2+^ sequestration or removal from the cytoplasm of pre-synaptic neurons, a problem that is associated with aging [56–58]. Other studies have shown that deletion of either pre-synaptic Ca^2+^ regulatory protein synaptotagmin IV or scaffolding protein RIM1ɑ leads to increased paired-pulse facilitation and exhibits deficits in contextual fear conditioning [59–61]. Overall, while the mechanisms underlying this pre-synaptic alteration in ischemic animals remains undetermined, the data presented are consistent with the hypothesis that pre-synaptic Ca^2+^ dysregulation may develop following CA, where increased synaptic Ca^2+^ over time may lead to increased synaptic dysfunction; future studies should investigate this connection.

Previous studies have suggested that various degrees of synaptic transmission dysfunction (primarily deficits in LTP) are evident 7 to 14 days following 10–12 min of global cerebral ischemia in young rats (2–3 month old) [52–54]; however, no discernable LTP deficit was observed in our study. Differences in the model used (ACA versus 2-VO or 4-VO), ischemia duration (6 min versus 10–12 min), or the age of the animals (9 months versus 3–4 months) may explain the lack of an impairment of LTP. Overall, these results may suggest that CA3 to CA1 synaptic transmission is dysfunctional beyond the initial mild ischemic insult (lasting at least seven days). However, additional experiments are still needed to fully investigate this synaptic transmission dysfunction following global cerebral ischemia.

Various studies have indicated that following cerebral ischemia, animals display numerous cognitive deficits [15, 28]. Here, our data on nine month old Fischer 344 rats suggests that there is a significant impairment or delay in memory formation, as there was an effect of surgery on the differences observed on the Barnes maze in both latency and distance measurements, as well as differences in spatial memory performance on the fear conditioning task. These results are in agreement with reports indicating spatial memory deficits following CA in both rats and mice [15, 33, 62]. Furthermore, we observed differences between ACA and sham groups in the use of a spatial search strategy, as well as the use of a systematic non-random search strategy (either serial or spatial). Although, there is some controversy as to the role of the hippocampus in the resulting spatial memory deficits [63, 64], our findings indicated extensive CA1 damage and spatial memory deficits, in agreement with a wide body of literature [15, 16, 65–67]. A potential explanation of the observed spatial memory deficits may be due to damage to the network connecting the medial prefrontal cortex and hippocampus [68]. It should be noted that this impairment was not due to a decrease in animal locomotor activity, as there was no significant difference in activity found in an open field test between sham and ACA animals (**S1 Fig**). While locomotor activity following CA has been controversial [33, 69], these differences in activity could be dependent on the duration or model of global ischemia and the strain of animal used.

The cognitive dysfunction observed 7 days following mild ACA in the Barnes circular platform maze and fear conditioning is suggestive of the synaptic transmission dysfunction and decreased number of non-compromised neurons observed in our electrophysiological and histological experiments. The data are in agreement with de la Tremblaye and Plamondon, (2011), who used 10–12 month old Wistar rats and 10 minutes of global cerebral ischemia (4-VO) to observe spatial memory deficits using the Barnes circular platform maze one week and contextual fear conditioning three weeks following ischemia [62]. Additionally, the spatial memory deficits may be long lasting, as Kiryk *et al*. (2011) found that spatial memory deficits were sustained for up to six months following cardiac arrest [33]. It should be noted that hippocampal-dependent memory formation was only impaired and not completely inhibited, which is commonly observed in cardiac arrest survivors [11, 12, 14]. This impairment was indicative of the animal’s ability to encode and retrieve information about the spatial orientation within their environment over time during their Barnes circular platform maze trials. Our electrophysiology data was correlative with this observation as LTP was induced following tetanus, indicating that the ability for memory formation is present at seven days. Finally, due to the low survival rate of 38%, a possible limitation of this study may be that the animals that survived seven days were those with minimal ischemic damage, potentially minimizing the spatial memory and synaptic deficits observed. Interestingly, this outcome may closely resemble clinical paradigms, as the out-of-hospital CA survival rate is only 8–10% [2, 6].

In summary, our results indicate that following ACA in nine month old Fischer 344 rats, there is a significant reduction in the number of non-compromised hippocampal CA1 and subiculum neurons, dysfunction in paired-pulse facilitation, and impairment in spatial memory encoding or learning. Despite these impairments in memory, animals displayed the ability to form spatial memories over-time. Additional studies are still needed to identify these pre-synaptic alterations following ACA and to further identify long-term cognitive deficits that could ensue from prolonged synaptic transmission dysfunction.

## Supporting Information

S1 Fig Locomotor activity following Sham or ACA procedure. Average distance traveled per animal in one 30 minute trial in an open field chamber. Sham animals traveled an average distance of 1943 ± 100.2 cm compared to ACA animals which traveled 1644 ± 212.4 cm (p >0.05) (Sham n = 6 ACA n = 6).
(TIF)

S1 Table Search Strategy Analysis. Two separate analyses were run. The first analysis divided search strategies into spatial (Sp), or non-spatial (Non) strategies. ACA rats used a spatial less often than sham rats (Chi-Square, *p<0.05). The second analysis divided search strategies into a non-random systematic (sys) (serial + spatial), or random (ran) strategies. ACA rats used a non-random systematic strategy less frequently than sham rats (Chi-Square, ***p<0.005). Sp = spatial, non = non-spatial (serial + random), Sys = systematic (serial + spatial), ran = random. * = p<.05, ** = p<0.01.
(TIF)
