# Supplementary figures and images for: Effect of Cardiac Arrest on Cognitive Impairment and Hippocampal Plasticity in Middle-Aged Rats
Source: PLoS One. 2015 May 1;10(5):e0124918. doi: 10.1371/journal.pone.0124918 (PMC4416883; doi:10.1371/journal.pone.0124918)

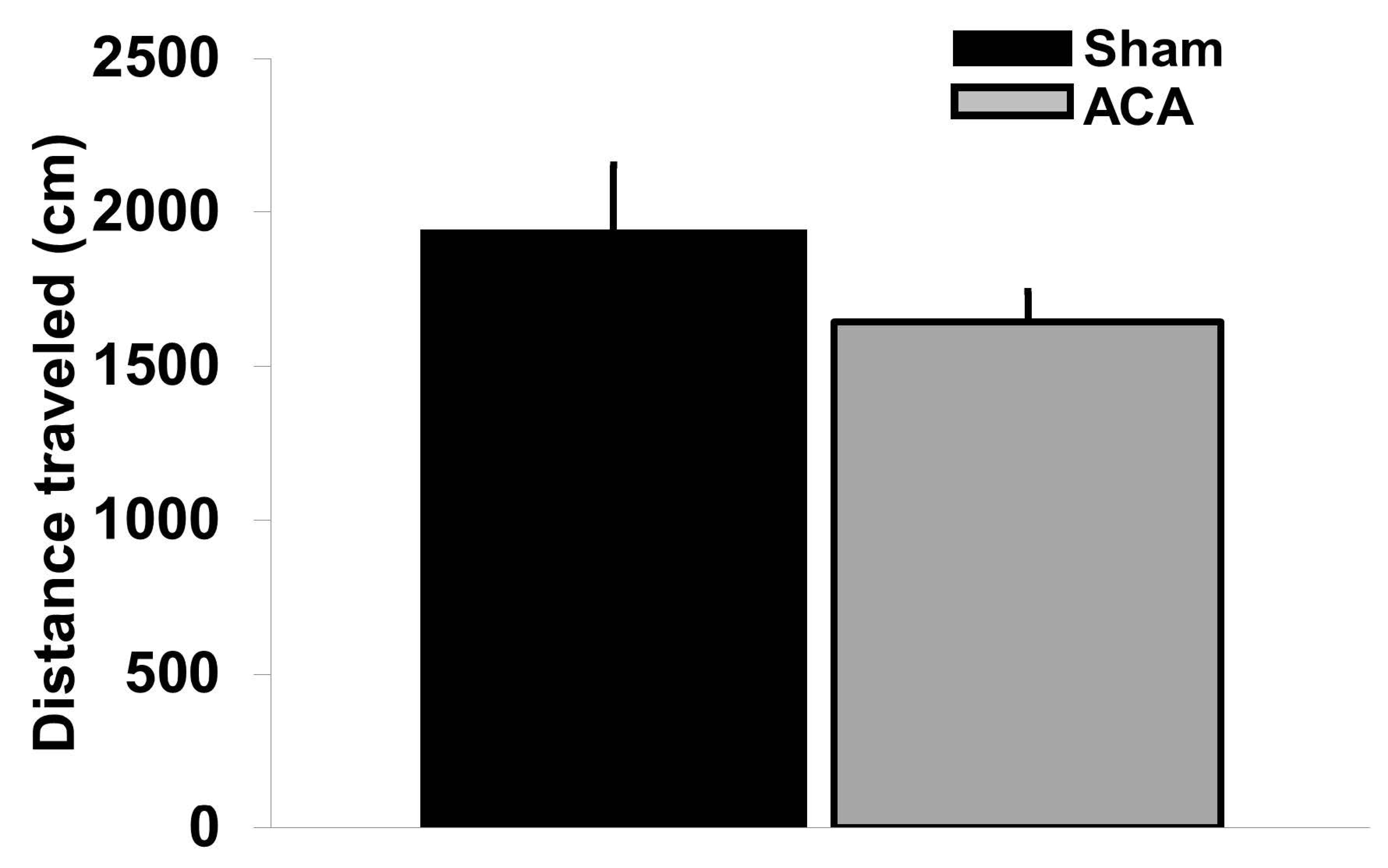

Supplement: S1 Fig — Average distance traveled per animal in one 30 minute trial in an open field chamber. Sham animals traveled an average distance of 1943 ± 100.2 cm compared to ACA animals which traveled 1644 ± 212.4 cm (p >0.05) (Sham n = 6 ACA n = 6). (TIF) [file pone.0124918.s001.tif]

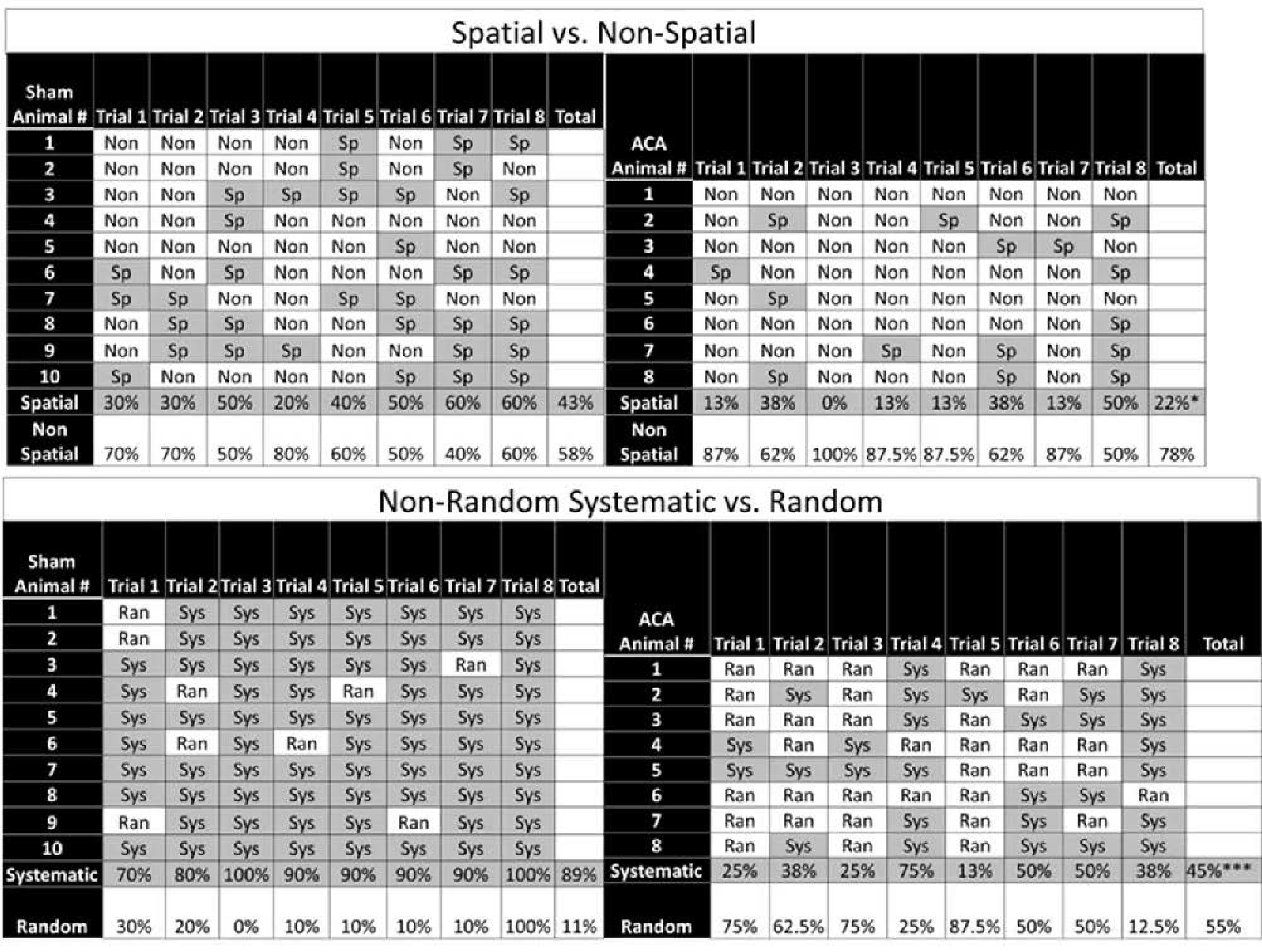

Supplement: S1 Table — Two separate analyses were run. The first analysis divided search strategies into spatial (Sp), or non-spatial (Non) strategies. ACA rats used a spatial less often than sham rats (Chi-Square, *p<0.05). The second analysis divided search strategies into a non-random systematic (sys) (serial + spatial), or random (ran) strategies. ACA rats used a non-random systematic strategy less frequently than sham rats (Chi-Square, ***p<0.005). Sp = spatial, non = non-spatial (serial + random), Sys = systematic (serial + spatial), ran = random. * = p<.05, ** = p<0.01. (TIF) [file pone.0124918.s002.tif]
